# Validation of diabetes mellitus and hypertension diagnosis in computerized medical records in primary health care

**DOI:** 10.1186/1471-2288-11-146

**Published:** 2011-10-28

**Authors:** Carmen de Burgos-Lunar, Miguel A Salinero-Fort, Juan Cárdenas-Valladolid, Sonia Soto-Díaz, Carmen Y Fuentes-Rodríguez, Juan C Abánades-Herranz, Isabel del Cura-González

**Affiliations:** 1 Unidad de Epidemiología Clínica e Investigación, Hospital Carlos III, (C/Sinesio Delgado, 10), Madrid, (28029), Spain; 2 Fundación de Investigación Biomédica, Hospital Carlos III, (C/Sinesio Delgado, 10), Madrid, (28029), Spain; 3 Unidad de Apoyo Técnico, Gerencia Adjunta de Planificación y Calidad del Servicio Madrileño de Salud, (C/O'Donell, 55), Madrid, (28007), Spain; 4 Gerencia, Hospital Carlos III, (C/Sinesio Delgado, 10), Madrid, (28029), Spain; 5 Unidad de Docencia e Investigación, Gerencia Adjunta de Planificación y Calidad del Servicio Madrileño de Salud, (C/Espronceda, 24), Madrid, (28003), Spain; 6 Departamento de Medicina Preventiva y Salud Pública, Universidad Rey Juan Carlos, (Avenida de Atenas s/n), Alcorcón, (28922), Spain

## Abstract

**Background:**

Computerized Clinical Records, which are incorporated in primary health care practice, have great potential for research. In order to use this information, data quality and reliability must be assessed to prevent compromising the validity of the results.

The aim of this study is to validate the diagnosis of hypertension and diabetes mellitus in the computerized clinical records of primary health care, taking the diagnosis criteria established in the most prominently used clinical guidelines as the gold standard against which what measure the sensitivity, specificity, and determine the predictive values.

The gold standard for diabetes mellitus was the diagnostic criteria established in 2003 American Diabetes Association Consensus Statement for diabetic subjects. The gold standard for hypertension was the diagnostic criteria established in the Joint National Committee published in 2003.

**Methods:**

A cross-sectional multicentre validation study of diabetes mellitus and hypertension diagnoses in computerized clinical records of primary health care was carried out. Diagnostic criteria from the most prominently clinical practice guidelines were considered for standard reference.

Sensitivity, specificity, positive and negative predictive values, and global agreement (with kappa index), were calculated. Results were shown overall and stratified by sex and age groups.

**Results:**

The agreement for diabetes mellitus with the reference standard as determined by the guideline was almost perfect (κ = 0.990), with a sensitivity of 99.53%, a specificity of 99.49%, a positive predictive value of 91.23% and a negative predictive value of 99.98%.

Hypertension diagnosis showed substantial agreement with the reference standard as determined by the guideline (κ = 0.778), the sensitivity was 85.22%, the specificity 96.95%, the positive predictive value 85.24%, and the negative predictive value was 96.95%. Sensitivity results were worse in patients who also had diabetes and in those aged 70 years or over.

**Conclusions:**

Our results substantiate the validity of using diagnoses of diabetes and hypertension found within the computerized clinical records for epidemiologic studies.

## Background

In recent decades, Computerized Clinical Records (CCR) are being used in the routine medical practice of primary health care (PHC) in the Spanish National Health System (NHS). Indeed, by 2007, 98.8% of general practices were computerized and 88% of the population had a primary care electronic health records [1]. There are many software systems to manage the CCR. In Spain the most frequently implemented CCR is the OMI-AP^® ^program.

Other countries like Canada, the United States of America, and some European Countries have a significant experience using computerized health databases obtained with PHC records [2-4]. The most widely used is the GPRD (General Practice Research Database), which contains information introduced prospectively since 1987 by more than 1,500 general practitioners (GPs) in the PHC of the United Kingdom and includes 7% of the population [5]. The GPRD has been widely used for research studies, with over 700 associated papers published up to date in peer-reviewed journals [5].

Electronic Health Records provide great potential for research, because of their ability to provide data for large populations. Even though the CCR can be used for research, it is important to note that the data are collected primarily for routine clinical rather than for researching purposes. Data quality and reliability must be assessed by researchers who use the resources found in the CCR in order to prevent compromising the results.

There are several approaches found in the literature but there is not an agreed upon standard approach to evaluate the quality and accuracy.

Related validation studies have attempted to show whether the cases with diagnostic codes indeed have that condition. Two recent systematic reviews of validation studies within the GPRD have shown that most (90%) of the coded diagnoses, from many diseases, are 'validated'[6,7]. In order to perform these validations, 83% of them used sources of information external to the GPRD, mostly questionnaires from GPs, hospital reports, copies of the Medical history or comparisons with disease rates obtained from other registries.

The validation of the diagnosis information included in BIFAP (Base de Datos para la Investigación Farmacoepidemiológica en Atención Primaria), population-based database in Spain containing information of more than 2.5 million patients, was carried out by sending questionnaires to collaborating doctors requiring hospital reports and other information in paper format of the clinical history and doctor's activities reports [8]. External validations were also carried out comparing the results with other sources of information, such as the national health survey and the death registry.

In order to guarantee the quality of the studies performed with the data from the CCR and because few studies have evaluated the quality of those registries, it is necessary to check their validity in our setting. The abovementioned methodology, that used the medical records of the second health care level as the standard reference, does not seem appropriate for chronic diseases such as hypertension (HTN) and diabetes mellitus (DM), which mainly were diagnosed and followed up in the PHC.

The aim of this study is to validate the diagnosis of HTN and DM coded in the CCR of PHC, taking the diagnosis criteria established in the most prominently used clinical guidelines as the gold standard.

## Methods

### Design

Cross-sectional validation study of the diagnoses of DM and HTN in the CCR of PHC.

### Setting

The study was carried out in the 21 health centers of the health area 4, in the northeast urban zone of the Community of Madrid, which serves a population of 777,426 people. All health centers have computerized patient records since at least for 10 years.

### Sources of information

The CCR administered by the software OMI-AP^® ^were structured around a list of episodes (problems in the bio-psycho-social sphere, reasons for consultation, etc). The episodes are listed with an alphanumeric code, which corresponds to the International Classification of Primary Care (ICPC), and a description or clinical label. The same code can be described by using many different clinical labels.

It is possible to link these episodes with diagnostic tests, prescriptions, protocols, therapeutic interventions, referrals, temporary incapacity to work reports, and free-text annotations. The laboratory results are automatically recorded in the CCR.

In PHC, text and codes are entered by the GPs during clinical care, as part of their routine clinical practice. The CCR incorporates a user-friendly instrument to encode episodes in order to make it acceptable and useful to the GPs, who are not professional coders. This instrument is a search system based on clinical labels that assigns the code automatically. The program allows the modification of the descriptions, but not the code which could be substituted or erased if deemed necessary.

Data extraction was conducted using the ICPC code from the CCR of patients.

### Inclusion and exclusion criteria

The study population comprised those patients who met the following inclusion criteria: had at least one record within the CCR in the health centers of health area 4 as of January 1^st ^2010; over 18 years of age; had an ICPC code in their CCR corresponding to DM (32,377 patients with code T90) or to HTN (91,065 patients with codes K86 or K87), respectively.

Patients were not included if they met any of the following exclusion criteria: had not at least one plasma glucose measurement (7.3%) or two Blood pressure (BP) measurements (22.9%) in their CCR for the validation of DM and HTN, respectively.

### Samples

Given the absence of reference information over the proportion of incorrectly classified cases (false negatives or false positives), the maximum indetermination was assumed (p = (1-p) = 0.5).

With this assumption, and in order to obtain a confidence of 95% and a precision of 5%, the required sample size was 384 patients for each variable. We increased it up to 423 to adjust for a foreseeable loss of 10% between the sampling and validation of the diagnosis (change of address, death or other reasons).

Four different samples of patients were obtained: with DM code (sample 1), without DM code (sample 2), with HTN code (sample 3), and without HTN code (sample 4) in the CCR. The first two were used to validate the episodes of DM and the latter two were used to validate the episodes of HTN.

Samples 1 and 3 were obtained by simple random way whilst samples samples 2 and 4 were obtained by individual matching by age and sex with samples 1 and 3, respectively.

### Methods

Diagnostic test aims to correctly classify patients and healthy for a disease or clinical condition. The validation of a diagnostic test is performed by comparing their results, both positives and negatives, with those obtained by the best instrument for measuring the phenomenon under study (gold standard).

In this study, documented diagnosis of DM and HTN were considered as the diagnostic tests. In order to perform the validation, they were compared against the gold standards.

The gold standard for DM were the diagnostic criteria established in 2003 American Diabetes Association Consensus Statement for diabetic subjects, that that were still in effect in 2010 [9]. The reference diagnostic criteria for DM were shown in Table 1.

**Table 1:** Diabetes Mellitus diagnostic criteria.

**Diabetes mellitus diagnostic criteria**

• Fasting plasma glucose ≥ 126 mg/dl (7.0 mmol/l).
• Symptoms of hyperglycemia (polyuria, polydipsia, and unexplained weight loss) and a casual (random) plasma glucose ≥ 200 mg/dl (11.1 mmol/l).
• 2-h plasma glucose ≥ 200 mg/dl (11.1 mmol/l) during the 75 g oral glucose tolerance test.
• On therapy for Diabetes mellitus and previous diagnosis of Diabetes Mellitus in medical records.
• Gestational diabetes mellitus:
- To make a diagnosis of Gestational diabetes mellitus, at least two of the following plasma glucose values must be found in the 100 g oral glucose tolerance test:
■ Fasting plasma glucose: ≥ 95 mg/dl
■ 1 h: ≥ 180 mg/dl
■ 2 h: ≥ 155 mg/dl
■ 3 h: ≥ 140 mg/dl.
- On therapy for Diabetes mellitus and diagnosis of Gestational diabetes mellitus in medical records.

* In the absence of unequivocal hyperglycemia, these criteria should be confirmed by repeat testing on a different day.

The gold standard for HTN were the diagnostic criteria established in the Joint National Committee (JNC 7) of the United States published in 2003, which were shown in Table 2[10].

**Table 2:** Hypertension diagnostic criteria.

**Hypertension diagnostic criteria**

• Average of two or more properly measured, systolic blood pressure readings on each of two or more office visits ≥ 140 mmHg (≥ 130 mmHg for patients with diabetes and chronic kidney disease)
• Average of two or more properly measured, diastolic blood pressure readings on each of two or more office visits ≥ 90 mmHg (≥ 80 mmHg for patients with diabetes and chronic kidney disease)
• On therapy with antihypertensive medications and diagnosis of Hypertension in medical records.

These criteria agree with recommendations given by other major scientific societies, such as the World Health Organization [11], the European Society for the Study of Diabetes (EASD) [12], the European Society of Hypertension (ESH) [13,14], the European Society of Cardiology [14], the Canadian Diabetes Association [15] and with the recommended guidelines in Spain [16-19].

Subjects of samples 1 and 2 were considered with DM as long as they fulfilled at least one of the criteria described in Table 1. Subjects from samples 3 and 4 that fulfilled any criteria from Table 2 were considered patients with HTN.

**Figure 1:**
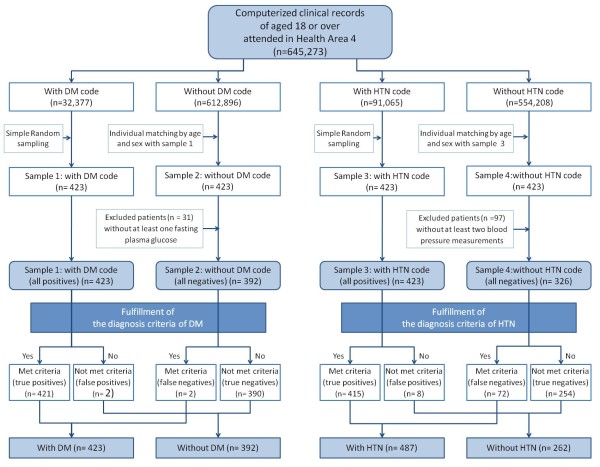
**Validation algorithm**. DM: diabetes mellitus; HTN: hypertension.

We consulted the computerized medical records of patients to verify concordance with the above criteria. The validation algorithm is shown in the Figure 1.

The evaluation team consisted of three general practitioners, with experience using the OMI-AP program. We conducted a peer evaluation with two reviewers and a third evaluator who resolved discrepancies.

### Statistical Analysis

A descriptive analysis of the study population and samples was carried out. The age was expressed by means of average percentiles 25 and 75, and the qualitative variables were summarized with their relative frequency.

Sensitivity (Sn), specificity (Sp), positive predictive values (PPV) and negative predictive values (NPV) were calculated, with their confidence intervals at 95% (CI), overall and stratified by gender, age group and DM.

In addition, a sensitivity analysis was performed determining the relative impact on predictive values of varying assumptions regarding the prevalence of DM and HTN.

The Sn is the proportion of cases with DM or HTN codes in the CCR among those that fulfilled the diagnosis criteria of Tables 1, respectively. The Sp is the proportion of cases without DM or HTN code in the CCR among those that did not fulfill the diagnosis criteria. Positive predictive value is the probability that people with DM or HTN code in the CCR meet the diagnostic criteria, while negative predictive value is the probability that people without DM or HTN code do not meet the criteria.

We checked whether the Sn and Sp were different by gender, age group and DM Through the homogeneity test based on a χ^2 ^statistic. In case that the application conditions of the test were not met (any expected cell count under 5), then the Fisher's exact bilateral test was used.

When diagnostic tests are applied to the population, the proportion of those testing positive (apparent prevalence) can not be used as an estimation of the prevalence of a disease in that population, because the Sn and Sp of these tests are usually less than 100%. Thus, the proportion of individuals with a positive result includes false positive cases and excludes cases that are false negatives, so in order to estimate the true prevalence of a disease from diagnostic tests; it is required to adjust for the misclassification resulting from the Sn and Sp of the used test. In this study we have used the formula proposed by Rogan and Gladen for this adjustment [20].

$$\text{True prevalence }=\;\left(\text{apparent prevalence }+\text{ Sp }-\text{ 1}\right)\;∕\;\left(\text{Sn }+\text{ Sp }-\text{1}\right)$$

The degree of overall agreement between the registered diagnosis and the reference standard, as well as the inter-observer agreement, was determined by the kappa index with their CI. According to this value, the agreement was considered slight (≤ 0.20), fair (0.21-0.40), moderate, (0.41-0.60), substantial (0.61-0.80) or almost perfect agreement (≥ 0.81) [21].

The statistic analysis of the information was performed with SPSS software (version 15.0, SPSS Inc., Chicago, Illinois), the CI of the kappa index and the predictive values were calculated with macros for SPSS of the Applied Statistics Laboratory in Universidad Autónoma de Barcelona: !KAPPA and !DT, respectively [22,23].

### Ethical Aspects

In order to ensure the confidentiality, the study was developed as stipulated in the Spanish Personal Data Protection Law. The protocol of the study was approved by the ethics committee of the Hospital Carlos III in Madrid and all of the evaluators signed a confidentiality clause.

## Results

The main demographic characteristics of the population of patients over 18 years old attended in the health area 4 with episodes of DM (ICPC T90) and HTN (ICPC K86 or K87) registered in the CCR, as well as the selected samples are described in Table 3.

**Table 3:** Main demographic characteristics of the population of patients over 18 years old attended in Health Area 4 and of samples.

	N (%)	Age (years) median (IQR)	Age ≥ 70 years (%)	Gender female (%)	DM (%)
**Patients with diagnosis code of diabetes (T-90)**	32,377 (5.02)	71.15 (60.03-78.95)	52.37	50.83	

**Sample of patients with diagnosis code of diabetes (sample 1)**	423 (1.31)	70.31 (60.22-78.20)	50.83	50.83	
**- Correct diagnosis (TP)**	421 (99.53)	70.31 (60.25-78.43)	50.83	50.83	
**- Incorrect diagnosis (FP)**	2 (0.47)	64.33 (51.22-77.44)	50	50	

**Patients without diagnosis code of diabetes (T-90)**	612,896 (94.98)	43.63 (33.27-60.08)	14.85	56.34	

**Sample of patients without diagnosis code of diabetes (sample 2)**	392 (0.06)	69.66 (59.57-78.05)	49.2	51.8	
**- Correct diagnosis (TN)**	390 (99.49)	69.66 (59.52-78.03)	49.2	51.8	
**- Incorrect diagnosis (FN)**	2 (0.51)	74.23 (62.79-85.67)	50	50	

**Patients with diagnosis code of hypertension (K-86 ó K-87)**	91,065 (14.11)	71.96 (61.66-79.41)	54.53	58.58	21.55

**Sample of patients with diagnosis code of hypertension (sample 3)**	423 (0.46)	72.63 (61.56-79.40)	57.92	58.63	24.59
**- Correct diagnosis (TP)**	415 (98.11)	72.67 (61.65-79.40)	58.31	58.55	24.82
**- Incorrect diagnosis (FP)**	8 (1.89)	64.00 (55.18- 76.86)	37.5	62.5	12.5

**Patients without diagnosis code of hypertension (K-86 or K-87)**	554,208 (85.89)	41.47 (32.21-54.68)	10.52	55.65	2.3

**Sample of patients diagnosis code of hypertension (sample 4)**	326 (0.06)	70.35 (61.77-79.27)	57.98	57.06	14.72
**- Correct diagnosis (TN)**	254 (77.91)	71.68 (61.15-78.26)	53.15	57.48	8.66
**- Incorrect diagnosis (FN)**	72 (22.09)	77.74 (69.32-83.99)	75	55.56	36.11

IQ: interquartile range; TP: true positive; FP: false positive; TN: true negative; FN: false negative; DM: diabetes mellitus.

The 7.3% of patients from sample 2 (without DM code) had to be excluded because there was not at least one fasting plasma glucose. They were 64.5% males with a mean age of 73.6 (SD 14.4) years. There were significant differences in mean age between patients excluded and not excluded.

The 22.9% of patients from sample 4 (without HTN code) had to be excluded because there were not at least two BP measurements. They were 36.1% males with a mean age of 69.1 (SD 11.6) years. There were no significant differences in mean age and female proportion between patients excluded and not excluded from the sample.

The prevalence of patients diagnosed with DM was 5.02%, slightly higher in males and in patients aged 65 years or older. The prevalence of patients with diagnosis of HTN was 14.11%, higher in women, in people aged 70 years or older and in patients with diagnosis of DM.

In our study, the diagnosis of DM was confirmed in 99.5% of the cases (sensitivity) and in 99.49% of those without diagnosis of DM did not meet the diagnosis criteria (specificity). There were no significant differences when stratifying by age groups or sex.

The sensitivity of the diagnosis of HTN was 85.22%, decreasing significantly in people over 69 years old (81.76%) and in patients with diagnosis of DM (79.85). The specificity of the diagnosis of HTN was 96.95%, with no significant differences when stratifying by sex or age groups (Table 4).

**Table 4:** Sensivity, specificity and agreement for diabetes mellitus and hypertension.

	TN	FN	FP	TP	Inter-observers agreement kappa (CI 95%)	Sensitivity % (CI 95%)	χ^2^ p-value	Specificity % (CI 95%)	χ^2^ p-value	Diagnostic agreement kappa (CI 95%)
**Diabetes**	390	2	2	421	0.988 (0.977 - 0.998)	99.53 (98.29 - 99.87)		99.49 (98.16 - 99.86)		0.990 (0.981 - 1)

**- Male**	188	1	1	207	0.985 (0.968 - 1)	99.52 (97.33 - 99.92)	1*	99.52 (97.34 - 99.92)	1*	0.990 (0.976 - 1)
**- Female**	202	1	1	214	0.990 (0.977 - 1)	99.54 (97.41 - 99.92)		99.53 (97.40 - 99.92)		0.990 (0.977 - 1)

**- Age < 70**	198	1	1	207	0.980 (0.961 - 1)	99.52 (97.33 - 99.92)	1*	99.50 (97.21 - 99.91)	1*	0.990 (0.977 - 1)
**- Age ≥ 70**	192	1	1	214	0.995 (0.985 - 1)	99.54 (97.41 - 99.92)		99.48 (97.12 - 99.91)		0.990 (0.977 - 1)

**Hypertension**	254	72	8	415	0.941 (0.916 - 0.967)	85.22 (81.79 - 88.09)		96.95 (94.09 - 98.45)		0.778 (0.732 - 0.823)

**- Male**	32	32	3	172	0.937 (0.897 - 0.978)	96.95 (94.09 - 98.45)	0.634	97.30 (92.35 - 99.08)	1*	0.770 (0.700 - 0.841)
**- Female**	40	40	5	243	0.944 (0.911 - 0.977)	85.87 (81.33 - 89.45)		96.69 (92.48 - 98.58)		0.783 (0.724 - 0.842)

**- Age < 70**	119	18	5	173	0.96 (0.928 - 0.992)	90.58 (85.6 - 93.96)	0.007	95.97 (90.91 - 98.27)	0.482*	0.850 (0.791 - 0.909)
**- Age ≥ 70**	135	54	3	242	0.926 (0.888 - 0.964)	81.76 (76.96 - 85.74)		97.83 (93.80 - 99.26)		0.724 (0.660 - 0.789)

**- Without DM**	232	46	7	312	0.947 (0.921 - 0.974)	87.15 (83.29 - 90.23)	0.045	97.07 (94.08 - 98.57)	0.526*	0.947 (0.921 - 0.974)
**- With DM**	22	26	1	103	0.882 (0.781 - 0.983)	79.85 (72.11 - 85.86)		95.65 (79.01 - 99.23)		0.522 (0.376 - 0.668)

* Fisher's exact bilateral test; TP: true positive; FP: false positive; TN: true negative; FN: false negative; CI 95%: confidence interval at 95%; DM: diabetes mellitus.

The degree of overall agreement between the diagnosis in the CCR and the standard of reference, measured as the kappa index, was almost perfect for DM (κ = 0.990), and substantial agreement for the HTN (κ = 0.778), as shown in Table 4. The worst result was obtained for HTN in the subgroup of patients with diagnosis of diabetes.

The degree of global inter-observers agreement, measured with the kappa index was very high, both for DM (κ = 0.988) and HTN (κ = 0.941), and for the different categories of sex, age groups and diagnosed diabetes. In all cases, the kappa index was higher than 0.880.

Table 5 shows the apparent prevalences (diagnosed in the CCR), true prevalences (fulfillment of the diagnosis criteria) as well as the positive and negative predictive values. The true prevalences were, for both diseases, higher than the apparent prevalences. The between them was 0.89% for the DM diagnosis and 21.45% for HTN, which increased up to 32.36% in patients diagnosed with diabetes. The probability that the diagnostic criteria could be confirmed in patients with diagnosed DM was 91.23% and 99.98% in HTN.

**Table 5:** Apparent prevalence (diagnosis in the CCR), true prevalence (fulfillment of the diagnosis criteria) and predictive values of diabetes and hypertension.

	Apparent prevalence * (CI 95%)	True prevalence ** (CI 95%)	PPV (CI 95%)	NPV (CI 95%)
**Diabetes**	5.02 (4.96 - 5.07)	5.06 (5.01 - 5.12)	91.23 (72.30 - 97.64)	99.98 (99.90 - 99.99)

**- Male**	5.61 (5.53 - 5.70)	5.67 (5.58 - 5.75)	92.59 (63.89 - 98.88)	99.97 (99.80 - 100)
**- Female**	4.55 (4.48 - 4.62)	4.59 (4.52 - 4.66)	91.11(59.19 - 98.64)	99.98 (99.84 - 100)

**- Age < 70**	2.89 (2.84 - 2.93)	2.91 (2.87 - 2.96)	85.58 (45.66 - 97.67)	99.99 (99.90 - 100)
**- Age ≥ 70**	15.72 (15.50 - 15.94)	15.87 (15.65 - 16.09)	97.32(83.69 - 99.61)	99.91 (99.38 - 99.99)

**Hypertension**	14.11 (14.03 - 14.20)	17.14 (17.05 - 17.23)	85.24 (74.46 - 91.96)	96.94 (96.24 - 97.52)

**- Male**	13.31 (13.18 - 13.43)	16.27 (16.13 - 16.41)	85.84 (66.47 - 94.88)	96.96 (95.87 - 97.78)
**- Female**	14.75 (14.63 - 14.86)	17.82 (17.70 - 17.95)	84.90 (70.34 - 93.02)	96.93 (95.94 - 97.68)

**- Age < 70**	7.75 (7.68 - 7.82)	8.91 (8.84 - 8.99)	68.72 (48.18 - 83.85)	99.05 (98.53 - 99.39)
**- Age ≥ 70**	46.07 (45.77 - 46.36)	57.86 (57.56 - 58.15)	98.10 (94.40 - 99.37)	79.61 (75.40 - 83.27)

**- Without DM**	11.66 (11.58 - 11.74)	13.81 (13.72 - 13.89)	82.52 (69.44 - 90.74)	97.94 (97.32 - 98.42)
**- With DM**	60.60 (60.07 - 61.14)	80.22 (79.78 - 80.65)	98.68 (91.62 - 99.80)	53.92 (45.09 - 62.52)

PPV: positive predictive value; NPV: negative predictive value; CI 95%: confidence interval at 95%.

Given that the PPV is directly proportional to the prevalence of the disease and the NPV is inversely proportional to the prevalence, we estimated the PPV and NPV for different true prevalences of HTN and DM (Table 6).

**Table 6:** Predictive values for hypertension and diabetes for different prevalences (sensitivity analysis).

Prevalence	Diabetes		Hypertension	
	
	PPV (CI 95%)	NPV (CI 95%)	PPV (CI 95%)	NPV (CI 95%)
**4%**	89.05 (67.11 - 97.01)	99.98 (99.92 - 100)	53.76 (36.99 - 69.72)	99.37 (99.22 - 99.49)

**4.5%**	90.19 (69.76 - 97.34)	99.98 (99.91 - 99.99)	56.80 (39.91 - 72.26)	99.29 (99.12 - 99.42)

**5%**	91.13 (72.04 - 97.61)	99.98 (99.90 - 99.99)	59.50 (42.59 - 74.42)	99.20 (99.02 - 99.36)

**5.5%**	91.91 (74.02 - 97.84)	99.97 (99.89 - 99.99)	61.89 (45.06 - 76.29)	99.12 (98.91 - 99.29)

**6%**	92.57 (75.76 - 98.02)	99.97 (99.88 - 99.99)	64.05 (47.36 - 77.91)	99.04 (98.81 - 99.22)

**6.5%**	93.13 (77.29 - 98.18)	99.97 (99.87 - 99.99)	65.99 (49.49 - 79.35)	98.95 (98.70 - 99.15)

**7%**	93.62 (78.66 - 98.32)	99.96 (99.86 - 99.99)	67.75 (51.47 - 80.62)	98.87 (98.60 - 99.08)

**7.5%**	94.05 (79.88 - 98.44)	99.96 (99.85 - 99.99)	69.35 (53.33 - 81.76)	98.78 (98.49 - 99.01)

**8%**	94.43 (80.98 - 98.54)	99.96 (99.84 - 99.99)	70.82 (55.07 - 82.78)	98.69 (98.38 - 98.94)

**8.5%**	94.77 (81.98 - 98.63)	99.96 (99.82 - 99.99)	72.17 (56.69 - 83.70)	98.60 (98.28 - 98.87)

**9%**	95.07 (82.88 - 98.72)	99.95 (99.81 - 99.99)	73.41 (58.22 - 84.54)	98.51 (98.17 - 98.80)

**9.5%**	95.34 (83.71 - 98.79)	99.95 (99.80 - 99.99)	74.55 (59.67 - 85.30)	98.42 (98.06 - 98.72)

**10%**	95.59 (84.47 - 98.86)	99.95 (99.79 - 99.99)	75.62 (61.03 - 86.00)	98.33 (97.94 - 98.65)

**11%**	96.02 (85.82 - 98.97)	99.94 (99.77 - 99.99)	77.53 (63.53 - 87.23)	98.15 (97.72 - 98.50)

**12%**	96.38 (86.97 - 99.07)	99.94 (99.74 - 99.98)	79.19 (65.77 - 88.29)	97.96 (97.49 - 98.35)

**13%**	96.68 (87.98 - 99.15)	99.93 (99.72 - 99.98)	80.66 (67.80 - 89.20)	97.77 (97.25 - 98.19)

**14%**	96.95 (88.85 - 99.22)	99.92 (99.69 - 99.98)	81.96 (69.64 - 90.00)	97.58 (97.02 - 98.04)

**15%**	97.18 (89.63 - 99.28)	99.92 (99.67 - 99.98)	83.12 (71.32 - 90.70)	97.38 (96.77 - 97.87)

**20%**	97.99 (92.45 - 99.49)	99.88 (99.53 - 99.97)	87.46 (77.89 - 93.25)	96.33 (95.49 - 97.02)

**25%**	98.49 (94.23 - 99.62)	99.84 (99.37 - 99.96)	90.29 (82.45 - 94.85)	95.16 (94.08 - 96.06)

**30%**	98.82 (95.45 - 99.70)	99.80 (99.20 - 99.95)	92.28 (85.80 - 95.95)	93.87 (92.51 - 94.99)

**35%**	99.06 (96.35 - 99.76)	99.75 (98.99 - 99.94)	93.76 (88.36 - 96.75)	92.41 (90.77 - 93.78)

**40%**	99.24 (97.03 - 99.81)	99.68 (98.75 - 99.92)	94.90 (90.38 - 97.36)	90.77 (88.81 - 92.42)

**45%**	99.38 (97.56 - 99.84)	99.61 (98.47 - 99.90)	95.80 (92.02 - 97.84)	88.91 (86.61 - 90.85)

**50%**	99.49 (98.00 - 99.87)	99.53 (98.14 - 99.88)	96.54 (93.37 - 98.22)	86.77 (84.11 - 89.04)

PPV: positive predictive value; NPV: negative predictive value; CI 95%: confidence interval at 95%.

## Discussion

The results of the study show a very high agreement of the diagnosis of DM in the CCR with the gold standard, and also a high sensitivity and specificity of the diagnosis of DM. The information obtained from the CCR provides a good estimation of the true prevalence of the illness, overall and in each category of sex and age groups.

For HTN, there was also a good overall agreement of the diagnosis in the CCR with the gold standard, high Sp and Sn, but lower than for diabetes. In patients with DM subgroup, the agreement was strikingly lower especially at the expense of Sn, because the under-diagnosis of HTN is much higher and the NPV, very influenced by the prevalence, is ostensibly lower. Similar results, of less magnitude, are found in the subgroup of patients aged 70 or over.

In our study the DM diagnosis was confirmed in 99.53% of the cases and HTN in 98.11%.

The systematic review of GPRD validation studies by Herrett and cols [6] shows that the different diagnoses studied were confirmed in 89% of the cases, although DM and HTN were not validated. Only a small proportion of the studies provided quantitative estimates of validity such as sensitivity and specificity.

We have not found published studies that used similar methodology to ours for the validation of HTN and DM diagnoses. For this reason, our results can only be compared with most similar validation studies, which used self-reported diagnosis by patients compared to biometric measures as reference standards.

The Sn obtained in our study for the diagnosis of DM (99.53%) is much higher than 69.7% they found in the DINO study [24], which validates the self-reported diagnosis of diabetes, HTN and hyperlipidemia of a population of 20 years and older in southern Spain. Published studies in other countries, also performed with self-reported diagnosis, present great heterogeneity, with Sn of DM between 58.9% in a Dutch study [25] and 85.2% in Taiwan [26].

Regarding the diagnosis of HTN, our Sn was a little bit lower (85.22%). In Spain, the DINO study estimated the Sn in 49.4% [24], in a subsample of the SUN study the Sn was 90.3% [27], and the EPIC Murcia cohort study obtained a sensitivity of 63.5% [28], taking medical records as the reference standard. In other countries, we found values that oscillate between 34.5% in the Dutch study aforementioned [25], and 82% found in a North-American study [29].

The high specificity obtained for the diagnosis of DM (99.49%) is consistent with the findings of other published studies, where the Sp is situated between 95.2% [30] and 99.6% [24]. The Sp obtained for the HTN (96.95%) is slightly above what was found by other authors, which oscillated between 80% [31] up to 96.8% [24].

The agreement found between the diagnosis of DM registered in the CCR and the fulfillment of the diagnosis criteria were very good (k = 0.990), above the one obtained in the DINO study (K = 0.78) [24].

For HTN, the agreement (k = 0.778) was lower than for diabetes, but higher than those found in the DINO study (k = 0.51) [24], EPIC-Murcia (k = 0.58) [28] and in the SUN (k = 0.66) [27].

The validation indexes obtained in our study are higher than those found by other authors, possibly because we have checked the diagnosis done by physicians and not those self-reported by patients.

Our study confirms the hypothesis found in other publications in which DM diagnosis has higher validity than HTN diagnosis [25,30,32]. This could be related to the perceived higher seriousness of physicians for DM than for HTN, and due to the DM diagnostic criteria that were changed less frequently and more uniform than those for HTN. In general, the parameters of validity found enable us to realize a precise estimation of the prevalence of diabetes [33] but a sub-estimation of the prevalence of HTN.

The under-diagnosis of HTN is a well-known phenomenon whose magnitude varies greatly in published studies. In a systematic review of 44 studies from different countries published in 2009 [34], the proportion of undiagnosed HTN on a worldwide level was estimated to be 46.2% for men and 58.5% for women. Different Spanish studies offer results from 14.9% in Navarra [27], to 49.4% in Galicia [35] and 31.74% obtained in the PREDIMERC study in Madrid [36]. In our study, we found 21.45% in global and 32.36% in the subgroup of patients with diabetes.

Very little is known about the prevalence of undiagnosed HTN in diabetic patients. In Spain, the DIAPA study found that 56.8% of patients with type 2 DM had BP > 130/85 mmHg, even though they were not previously diagnosed with HTN [37]. The lack of HTN diagnosis in patients with DM could be related to the cutoff values of diagnosis, which are lower for these patients (BP ≥ 130/80 mmHg) [10], and it is possible that some GPs had been using the diagnostic criteria of the general population (BP ≥ 140/90 mmHg).

When stratifying by sex and age groups, there were significant differences in the Sn of HTN with worse results in patients with DM and in those over 69 years, despite the fact that these patients are subject to a large number of revisions and so there were more chances to detect HTN. These results are different than other studies [24,25,27,28,30,32], where better results were obtained in older and diabetic patients. One possible explanation could be that other studies were done through questionnaires with volunteer participants. The methods used may have resulted in a selection bias, since the patients who are more worried about their health and those who have worse perception of their health may be more predisposed to participate.

The true prevalence of DM in our study was 5.06%, which is very similar to the obtained with the BIFAP database (5.8%) [8] and in the Spanish National Health Survey (4.79%) [38] but lower than 8.1% in the PREDIMERC study in Madrid [36].

The true prevalence of HTN in our study (17.14%) is similar to those obtained with the BIFAP database (16.1%) [8] and in the Spanish National Health Survey (18.89%) [38] but also lower than the findings in other studies in Spain. These studies found about 35% in the adult population [39] and 29.3% in the PREDIMERC study [36].

This differences observed in the magnitude of the prevalences could be due to the age of the patients included in PREDIMERC [36]. The patient range age was between 30 and 74 whereas our study includes all those aged 18 and over. If we had selected people aged 30 or over in our database, the true prevalence would have been 6.87% for DM and 23.72% for HTN.

Moreover, PREDIMERC was undertaken with volunteers, with an overall response rate of 56.4%, which may have produced a selection bias as was mentioned before. Furthermore, we cannot assure that false positives were due to misdiagnosis, simply that the verification of diagnostic criteria could not be met. This may have led to an underestimation of the prevalence in our study.

The prevalence of HTN in diabetic patients obtained in our study (80.22%) is close to the highest found in the studies published, which oscillate between 50% and 84% [37,40].

The study presents some limitations. These are that the information included may not have been completely exhaustive. Because of this a potential selection bias may exist. This is particularly true in view of the proportion of adults in nursing homes, chronic disease hospitals, or those treated in private practice. In addition, as the health area 4 in Madrid covers only urban population, patients living in rural areas were not represented in this study.

Alternatively, more than 95% of citizens have public health coverage with the Spanish National Health System [41], so we suppose that the proportion of assigned persons in the health area 4 who are not included in our study is low.

The high proportion of patients without available information to perform the validation (7.3% had not at least one plasma glucose level and 22.9% had not two or more BP measurements) could be due to the fact that these patients do not usually go to health centers. At least one or two years of active data are basically required in order to include clinical patient records in a study.

In both the GPRD and the BIFAP, GPs who voluntarily participate in the projects are the ones that refer the information, possibly introducing a selection bias (e.g. the pattern of patients care can differ among volunteer physicians and those who are not volunteers). Furthermore, the response rate of physicians to questionnaires can be low, as occurred in BIFAP with the validation of Upper Gastrointestinal Bleeding with a rate of the 58.4% [8]. Our database is exhaustive, containing information from the entire population attended in the health area, and registered by all the professionals, avoiding the possibility of the mentioned biases.

Published studies related to validation are aimed to show whether cases with diagnostic codes indeed have that condition. The use of the PPV as the only measurement presents the inconvenience that it depends on the prevalence of the disease, as shown in Table 6. Another weakness of these validation studies is that, with a few exceptions, they do not address the question of false negatives, which are cases of the disease who have not received a diagnostic code. There were missed cases in which the GPs did not make the diagnosis or when made diagnosis but encoded it wrong [42]. We argue that in any validation study, PPV, NPV, Sn, and Sp should be identified, as far as possible.

The validation of the diagnosis of CCR of PHC has facilitated the detection of areas of improvement in the clinical practice, such as under-diagnosis of HTN with differential classification bias for patients who also suffers diabetes.

These findings can be used to alert clinicians of subgroups for which the interventions could be more beneficial.

The use of secondary sources of information stored in computerized databases enables access data from large populations. This can facilitate the rapid identification of patients for observational studies or inclusion in interventions and may reduce the time and resources needed to obtain results. This greater efficiency constitutes one of the main advantages for using the databases as epidemiological research tools.

## Conclusions

The results obtained in this validation enable the usage of both DM and HTN diagnoses codes of the computerized clinical records of PHC as a valid tool, which can be used with confidence to perform epidemiological studies.

However, the HTN diagnosis in the CCR has lower sensitivity than DM diagnosis, especially in diabetic patients. Therefore, in this group of patients, the code of HTN diagnosis in the CCR is not enough in order to detect people without HTN since there would be selected a high amount of false negative results.

## Competing interests

The authors declare that they have no competing interests.

## Authors' contributions

CBL conceived the study, participated in its design, performed the statistical analysis and drafted the manuscript. MASF participated in the design and drafted the manuscript. JCV participated in the design and coordinated the research group. JCAH, ICG, SSD drafted the manuscript and made substantial contributions to the analysis and interpretation. CYFR helped in the statistical analysis and drafted the manuscript. All authors read and approved the final manuscript.

## Pre-publication history

The pre-publication history for this paper can be accessed here:

http://www.biomedcentral.com/1471-2288/11/146/prepub
